# Evidence for the Intense Exchange of MazG in Marine Cyanophages by Horizontal Gene Transfer

**DOI:** 10.1371/journal.pone.0002048

**Published:** 2008-04-23

**Authors:** Michael J. Bryan, Nigel J. Burroughs, Edward M. Spence, Martha R. J. Clokie, Nicholas H. Mann, Samantha J. Bryan

**Affiliations:** 1 Department of Biological Sciences, University of Warwick, Coventry, United Kingdom; 2 Warwick Systems Biology Centre, University of Warwick, Coventry, United Kingdom; 3 Department of Infection, Immunity and Inflammation, University of Leicester, Leicester, United Kingdom; University of British Columbia, Canada

## Abstract

**Background:**

S-PM2 is a phage capable of infecting strains of unicellular cyanobacteria belonging to the genus *Synechococcus*. S-PM2, like other myoviruses infecting marine cyanobacteria, encodes a number of bacterial-like genes. Amongst these genes is one encoding a MazG homologue that is hypothesized to be involved in the adaption of the infected host for production of progeny phage.

**Methodology/Principal Findings:**

This study focuses on establishing the occurrence of *mazG* homologues in other cyanophages isolated from different oceanic locations. Degenerate PCR primers were designed using the *mazG* gene of S-PM2. The *mazG* gene was found to be widely distributed and highly conserved among *Synechococcus* myoviruses and podoviruses from diverse oceanic provinces.

**Conclusions/Significance:**

This study provides evidence of a globally connected cyanophage gene pool, the cyanophage *mazG* gene having a small effective population size indicative of rapid lateral gene transfer despite being present in a substantial fraction of cyanophage. The *Prochlorococcus* and *Synechococcus* phage *mazG* genes do not cluster with the host *mazG* gene, suggesting that their primary hosts are not the source of the *mazG* gene.

## Introduction

Unicellular cyanobacteria of the genera *Synechococcus* and *Prochlorococcus* are abundant in the world's oceans, whilst phages infecting these organisms are thought to determine host community structure and to divert the flow of fixed carbon within the microbial loop (for review see [1]. Phage S-PM2 is a marine myovirus originally isolated from the English Channel that infects strains of marine *Synechococcus* [2], [3]. It has a comparatively large genome (∼196 Kb) whose sequence was published in 2005 [2], encoding 239 open reading frames (ORFs), 20 of which share similarities with genes in their *Synechococcus* host including key photosynthesis genes. ORF136 of S-PM2 encodes a homologue of the bacterial *mazG* gene, for which two potential metabolic roles have been proposed. Galperin *et al*. [4] suggest that MazG homologues, which are found in all three domains of life, act as “house-cleaning” enzymes that hydrolyse potentially harmful non-canonical nucleotides that are produced as a by-product of metabolism. However, MazG has also been implicated as a regulator of programmed cell death in *Eschericia coli* [5]. MazG, *in vivo*, prevented the normal accumulation of guanosine 3',5'-bispyrophosphate (ppGpp) during the stringent response to amino acid starvation [5]. ppGpp acts as a global regulator in *E. coli,* causing a redirection of transcription in favour of genes important for starvation survival [6], although it has recently been shown to also control elongation during DNA replication in response to nutritional status [7]. In unicellular cyanobacteria, albeit not marine, ppGpp was demonstrated to accumulate under conditions of energy limitation [8] and nitrogen starvation [9], whereas phage infection interfered with this accumulation [10]. It has been suggested that the phage-encoded MazG operates to reduce the ppGpp pool in infected *Synechococcus* cells [11]. This reduction in the ppGpp pool could potentially alter the physiology of an infected cell, mimicking that of a cell replete with nutrients and thus optimizing the production of progeny phage by reactivating the pathways of macromolecular synthesis. As a first step towards testing this hypothesis, we examined the occurrence of MazG homologues in phage isolated from a variety of oceanic provinces.

## Results

**Figure 1:**
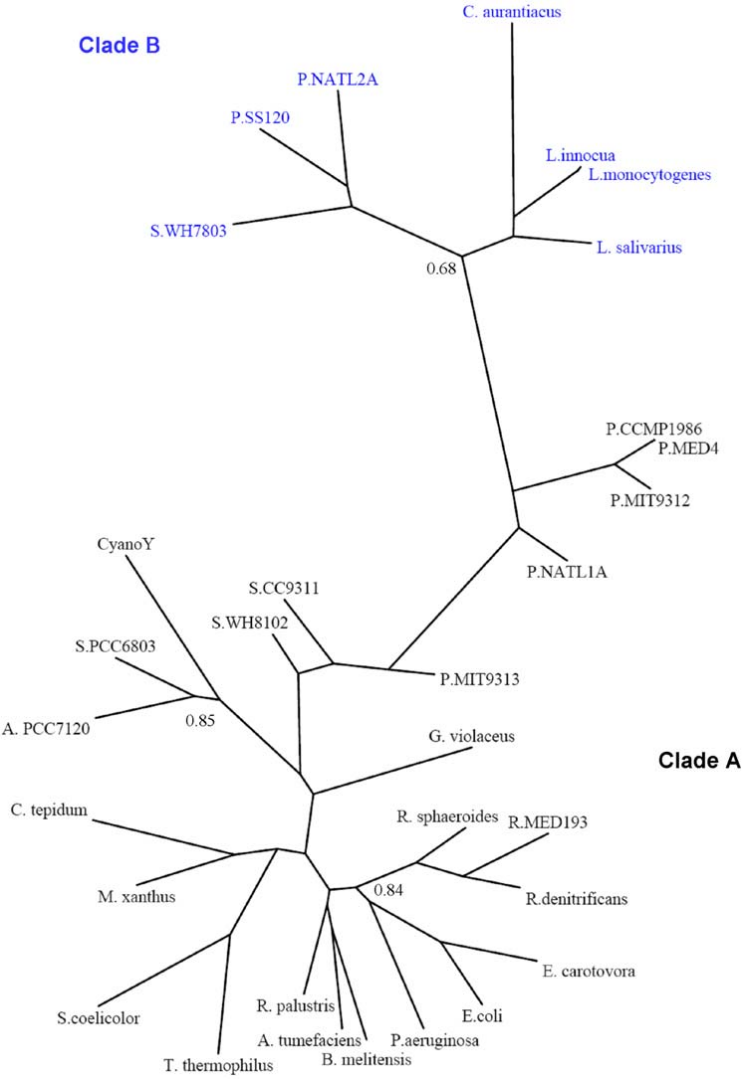
Consensus phylogenetic tree of the *mazG* gene for bacterial sequences. Species are: *Agrobacterium tumefaciencis*, *Anabaena* PCC7120, *Brucella melitensis, Chlorobium tepidum* TL5, *Chloroflexus aurantiacus* J-10-Fl, *Cyano Y* Yellowstone national Park, *Erwinia carotovora*, *Eschericia coli* K-12 W3110, *Gleobacter violaceus* PCC7421, *Lactobacillus salivarius*, *Listeria innocua* Clip11262, *Listeria monocytogenes* EGDe-, *Myxococcus xanthus* DK101, *Prochlorococcus sp*. CCMP1986, *Prochlorococcus sp*. MED4, *Prochlorococcus sp.* MIT9312, *Prochlorococcus sp.* MIT9313, *Prochlorococcus sp*. NATL1A *Prochlorococcus sp.* NATL2A, *Prochlorococcus sp*. SS120, *Pseudomonas aeruginosa*, *Rhodobacter sphaeroides* ATCC 17029, *Rhodopsedomonas palustris* CGA009, *Roseobacter denitrificans*, *Roseobacter sp.* MED193, *Streptomyces coelicolor* A3(2), *Synechococcus sp*. CC9311, *Synechocystis sp*. PCC6803, *Synechococcus sp.* WH7803, *Synechococcus sp.* WH8102, *Thermus thermophilus* HB27. Trees are unrooted and were generated from DNA codon alignments. The maximum likelihood tree has identical topology. Clade support values are shown at the nodes of the clades where support was less than 95%. Clade A (black) and Clade B (blue) refer to the two main clades of *mazG* observed in this study.

Initially, several species of bacteria were selected from KEGG and NCBI to represent all of the main phyla in the prokaryotic kingdom (Table 1) where a *mazG* homologue could be detected. Each species was selected on the following criteria: sequence similarity to the *mazG* gene from *E. coli* (where the role of MazG has been comparatively well characterised), and the presence of a MazG domain (pyrophosphohydrolase domain) in the homologue. Phylogenetic analysis using Mr Bayes (http://mrbayes.csit.fsu.edu/) was performed on the complete *mazG* gene sequences from these bacterial species (Figure 1), thereby determining key interrelationships and assisting in assessing host-phage relationships. Two distinct clades were identified from the trees. The first clade, **Clade A**, comprised *mazG* genes from freshwater, marine and terrestrial bacteria (in particular the proteobacteria) and the fresh water cyanobacteria. The proteobacteria comprise the γ, δ and α-proteobacteria and the α-Rhizobacteria. No detectable homologues of *mazG* in the β or ε-proteobacteria were detected. Marine cyanobacteria were also present in Clade A along with members from the deinococci, the actinobacteria and chlorobia.

**Table 1:** Bacteria and phage sequences used in this study, showing source databases and accession numbers.

Strain	Family	Source	Accession number	Clade
*E. coli* K-12 W3110	Gamma/enterobacteria	KEGG	AC 000091	A
*Rhodobacter sphaeroides* ATCC 17029	Alpha/others	KEGG	NC 009050	A
*Roseobacter* MED193	Alpha/others	NCBI	NZ AANB00000000	A
*Roseobacter denitrificans*	Alpha/others	KEGG	NC 068209	A
*Psedomonas aeruginosa* PAO1	Gamma/others	KEGG	NC 002516	A
*Erwinia carotovora*	Gamma/enterobacteria	KEGG	NC 004547	A
*Myxococcus xanthus* DK101	Gamma/delta	KEGG	N/A	A
*Brucella melitensis*	Alpha/rhizobacteria	KEGG	NC 003317	A
*Agrobacterium tumefaciens* C58	Alpha/rhizobacteria	KEGG	NC 003062	A
*Rhodopseudomonas palustris* CGA009	Alpha/rhizobacteria	KEGG	NC 005296	A
*Thermus thermophilus* HB27	Deinococcus-thermus	KEGG	NC 005835	A
*S. coelicolor*	Actinobacteria	KEGG	NC 003888	A
*Chlorobium tepidum* TL5	Green Sulphur Bacteria	KEGG	NC 002932	A
*Gleobacter violaceus* PCC7421	Cyanobacteria	Cyanobase	NC 005125	A
*Cyano Y*	Cyanobacteria	KEGG	NC 607775	A
*Anabaena sp.*PCC7120	Cyanobacteria	Cyanobase	BA 000019	A
*Synechocystis sp.* PCC6803	Cyanobacteria	Cyanobase	BA A17296	A
*Synechococcus sp*. WH8102	Cyanobacteria	Cyanobase	NC 005070	A
*Synechococcus sp*. CC9311	Cyanobacteria	KEGG	NC 008319	A
*Prochlorococcus marinus sp*. MIT9313	Cyanobacteria	Cyanobase	NC 005071	A
*Prochlorococcus marinus sp*. NATL1A	Cyanobacteria	KEGG	NC 008819	Aα
*Prochlorococcus marinus sp*. MIT9312	Cyanobacteria	KEGG	NC 007577	Aα
*Prochlorococcus marinus sp*. Pastoris	Cyanobacteria	KEGG	NC 005296	Aα
*Prochlorococcus marinus sp*. MED4	Cyanobacteria	Cyanobase	NC 005072	Aα
*Synechococcus sp*. WH7803	Cyanobacteria	NERC	NC 009481	B
*Prochlorococcus marinus sp*. SS120	Cyanobacteria	Cyanobase	NC 005042	B
*Prochlorococcus marinus sp*. NATL2A	Cyanobacteria	KEGG	NC 007335	B
*Lactobacillus salivarius*	Firmicutes/Lactobacilli	KEGG	NC 007929	B
*Listeria innocua*	Firmicutes/Bacillales	KEGG	NC 003212	B
*Listeria monocytogenes*	Firmicutes/Bacillales	KEGG	NC 003210	B
*Chloroflexus aurantiacus* ATCC 29366	Green non sulphur bacteria	NCBI	NZ AAAH00000000	B
Mx8 Myxobacteria phage	N/A	NCBI	NC 003085	-
L-5 Mycobacteria phage	N/A	NCBI	NC 001335	-
R.S101 Roseobacter phage	N/A	NCBI	NC 002519	-
P-SMM2 Prochlorococcus phage	N/A	NCBI	NC 006883	-
P-SMM4 Prochlorococcus phage	N/A	NCBI	NC 006884	-
Syn9 Synechococcus phage	N/A	NCBI	N/A	-

Clades are also shown as determined by the phylogenetic analysis (Figure 1). **Clade A** and **Clade B** refer to the two main clades of *mazG* observed in this study. Phage sequences taken from NCBI are shown at the bottom of the table. The sequenced phages all had the *mazG* gene.

**Figure 2:**
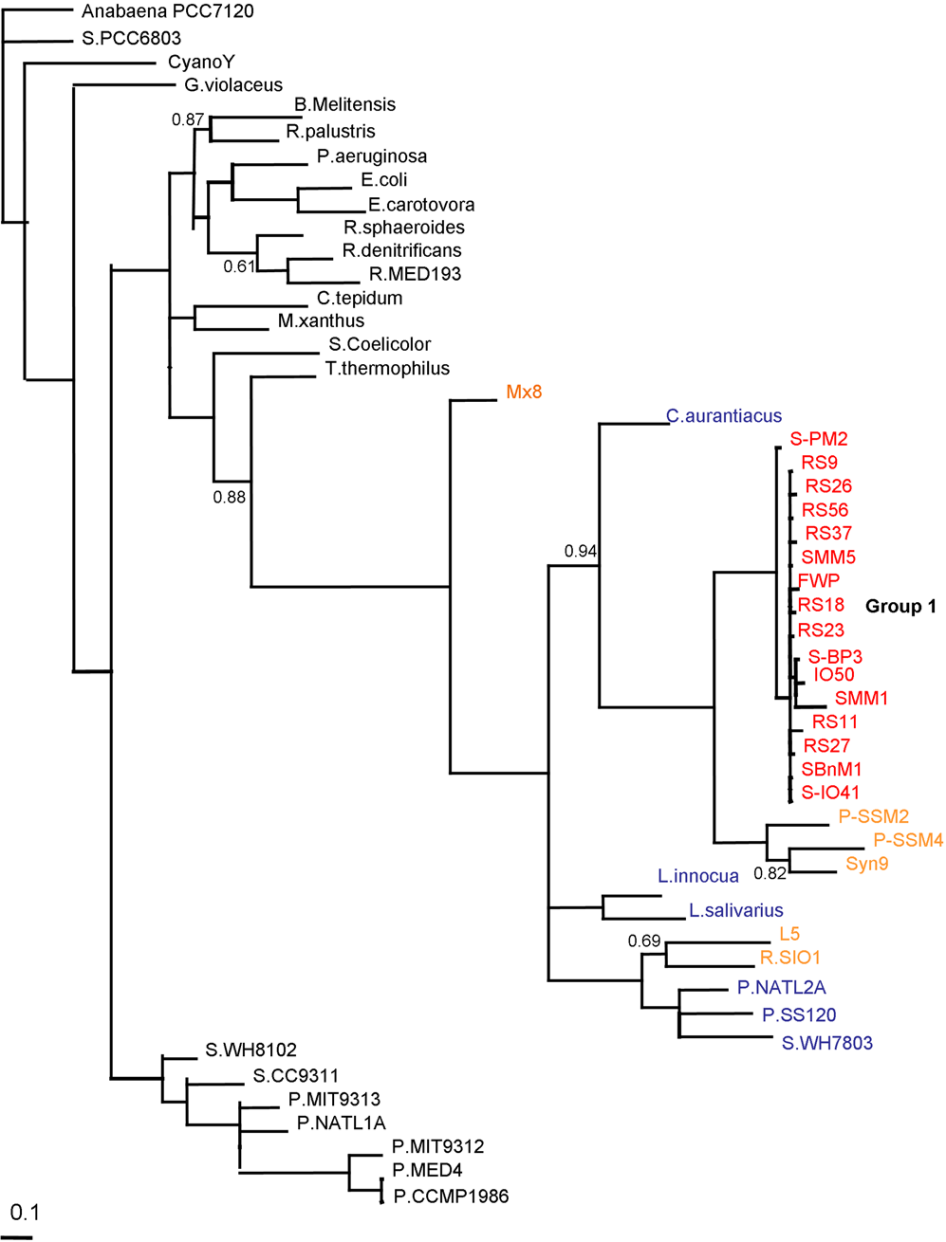
Consensus phylogenetic tree of the *mazG* gene from Group 1 phage isolates and bacteria. Bacteria sequences as in Figure 1 plus the following phage sequences: Group 1 phages S-PM2, S-IO41, IO50, S-BP3, S-MM1, SBnM1, RS23, FWP, RS18, RS56, S-MM5, RS37, RS26, RS27, RS9 and RS11 and 7 phage sequences from the NCBI database; Myxococcus phage Mx8, Roseobacter phage S101, Mycobacterium phage L-5, and cyanophages S-PM2, P-SMM4, P-SMM2 and Syn9. Trees are unrooted and were generated from DNA codon alignments. Clade support values are shown at the nodes of the clades, only values below 0.95 are shown. Group 1 is shown coloured in red, phage sequences from NCBI in orange, and bacteria as in Figure 1.

**Figure 3:**
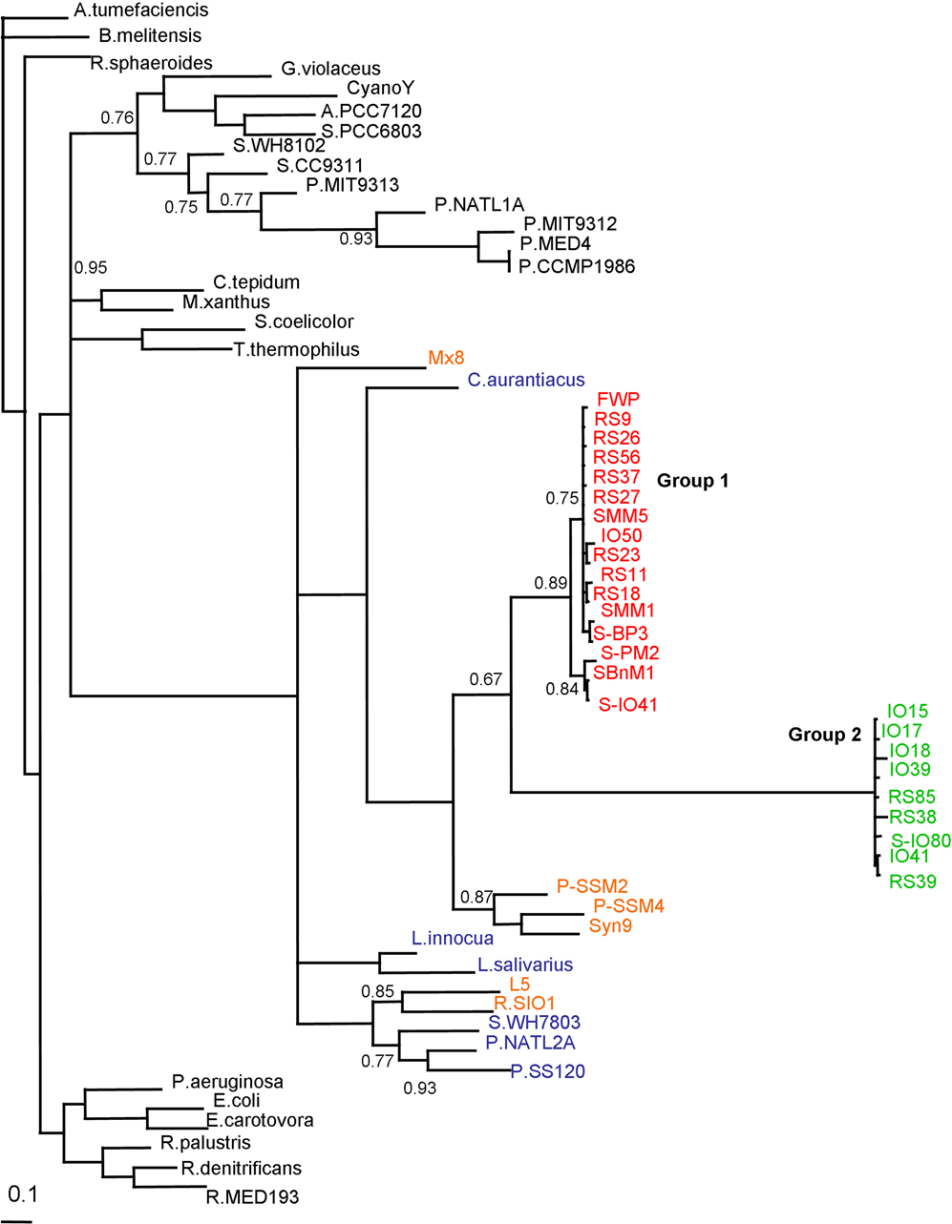
Consensus phylogenetic tree of the *mazG* gene from phage isolates and bacteria. Bacterial and phage sequences as before (Figure 2) with Group 2 phages IO41, RS85, IO39, RS39, IO15, S-IO80, IO18, RS38, and IO17. Trees are unrooted and were generated from DNA codon alignments. Clade support values are shown at the nodes of the clades, only values below 0.95 are shown. Colourings as in Figure 2 with the addition of Group 2 shown in green.

α These sequences were designated part of clade A despite being in the intervening arm in Figure 1 because they robustly grouped with Clade A in Figures 2,3.

The second clade, **Clade B**, is separated from Clade A by a combined branch length of over 1.0 (0.61, 0.08 and 0.42 as partitioned by common ancestors). This clade also contains terrestrial and marine bacteria, the marine cyanobacteria being on the interclade arm separating Clade A from Clade B. Clade B comprises the Chloroflexa, and the Firmicutes including the lactobacilli and bacillales, Table 1. Interestingly, marine cyanobacteria are represented in both clades, although they mainly occupy Clade B, away from the freshwater cyanobacteria in Clade A (Figure 1). The host for the phage S-PM2, *Synechococcus* WH7803, is located in Clade B. The presence of these two clades may indicate that the function of MazG in these bacteria is distinct, since the function of MazG has only been confirmed experimentally in *E. coli*.

DNA was extracted from Synechococcus phages isolated from sea water taken from a number of locations, including the Red Sea, Atlantic Ocean (Miami), the Indian Ocean, the North Sea, Bermuda, the Gulf of Mexico. One fresh water phage isolated from Lake Bourget, France, was also included (Table 2). PCR was carried out on each DNA sample using degenerate primers, (designed using Primer 3), to generate a product of 279 bp that corresponds to an internal portion of the *mazG* gene. A product of the correct size was detected for 41 out of the 84 phages screened. Products were excised from the agarose gel and cloned into a pGEM T-Easy vector, and then sequenced using the T7 forward primer. The resultant sequences were analysed using BlastN, to search for similar sequences in the NCBI database. PCR products from 15 out of the 41 phages showed 99% identity to the pyrophosphohydrolase *mazG* gene from S-PM2 (E value 8e-150); these, together with S-PM2, were defined as **Group 1** and comprise phages SBnM1, S-IO41, IO50, S-BP3, S-MM1, RS23, FWP (fresh water phage), RS18, RS56, S-MM5, RS37, RS26, RS27, RS9 and RS11. Each sequence was translated and Blast searches were carried out on the translated sequences using BlastP. All 16 phages demonstrated a particularly high similarity (E-value 0) to both phage and bacterial MazG proteins in the NCBI database. MotifScan was used to confirm the presence of the MazG domain, which defines the MazG protein. All 16 phage sequences were found to have a MazG domain present in the protein.

**Table 2:** Cyanophages categorised by their geographical location and whether the *mazG* gene was detected in the isolate.

Strain	Family	*Synechococcus* host strain(s)	Area of isolation	MazG detected by PCR primers	Source or Ref.	Translation
S-PM2	*Myoviridae*	WH7803, WH8012, WH8018	English Channel, Plymouth.	+	Wilson *et al.*1993	Yes
S-WHM1	*Myoviridae*	WH7803,WH8012	Woods Hole, Mass. USA	-	Wilson *et al.*1993	Yes
S-BM3	*Myoviridae*	WH7803, WH8103	Bermuda	-	Fuller *et al.*1998	Yes
S-BP3	*Podoviridae*	WH7803	Bermuda	+	Fuller *et al.*1998	Yes
SBnM1	*Myoviridae*	WH7803	Bergen, Norway	+	Wilson. 1994	Yes
S-MM1	*Myoviridae*	WH7803	Miami, Florida.	+	Wilson. 1994	Yes
S-MM4	*Myoviridae*	WH7803	Miami, Florida.	-	Wilson. 1994	Yes
S-MM5	*Myoviridae*	WH7803	Miami, Florida.	+	Wilson. 1994	Yes
S-PWM1	*Myoviridae*	WH7803	Gulf of Mexico	-	Suttle and Chan. 1993	Yes
S-PWM3	*Myoviridae*	WH7803, SYN48, SNC2, SNC1	Gulf of Mexico	-	Suttle and Chan. 1993	Yes
IO7	Unknown	α	Indian Ocean	-	Clokie *et al.*1999	No
IO8	Unknown	α	Indian Ocean	-	Clokie *et al.*2000	No
IO10	Unknown	α	Indian Ocean	+	Clokie *et al.*2001	Yes
IO12	Unknown	α	Indian Ocean	-	Clokie *et al.*2002	No
IO13	Unknown	α	Indian Ocean	-	Clokie *et al.*2003	No
IO14	Unknown	α	Indian Ocean	+	Clokie *et al.*2004	Yes
IO15	Unknown	α	Indian Ocean	+	Clokie *et al.*2005	No
IO17	Unknown	α	Indian Ocean	+	Clokie *et al.*2006	No
IO18	Unknown	α	Indian Ocean	+	Clokie *et al.*2006	Yes
IO19	Unknown	α	Indian Ocean	-	Clokie *et al.*2006	No
IO20	Unknown	α	Indian Ocean	-	Clokie *et al.*2006	No
IO21	Unknown	α	Indian Ocean	-	Clokie *et al.*2006	No
IO22	Unknown	α	Indian Ocean	-	Clokie *et al.*2006	No
IO23	Unknown	α	Indian Ocean	-	Clokie *et al.*2006	No
IO24	Unknown	α	Indian Ocean	-	Clokie *et al.*2006	No
IO25	Unknown	α	Indian Ocean	-	Clokie *et al.*2006	No
IO28	Unknown	α	Indian Ocean	-	Clokie *et al.*2006	No
IO31	Unknown	α	Indian Ocean	-	Clokie *et al.*2006	No
IO32	Unknown	α	Indian Ocean	-	Clokie *et al.*2006	No
IO33	Unknown	α	Indian Ocean	-	Clokie *et al.*2006	No
IO35	Unknown	α	Indian Ocean	-	Clokie *et al.*2006	No
IO37	Unknown	α	Indian Ocean	+	Clokie *et al.*2006	No
IO38	Unknown	α	Indian Ocean	+	Clokie *et al.*2006	No
IO39	Unknown	α	Indian Ocean	+	Clokie *et al.*2006	No
IO40	Unknown	α	Indian Ocean	+	Clokie *et al.*2006	Yes
IO41	Unknown	α	Indian Ocean	+	Clokie *et al.*2006	Yes
IO42	Unknown	α	Indian Ocean	-	Clokie *et al.*2006	No
IO43	Unknown	α	Indian Ocean	+	Clokie *et al.*2006	No
IO44	Unknown	α	Indian Ocean	+	Clokie *et al.*2006	Yes
IO45	Unknown	α	Indian Ocean	-	Clokie *et al.*2006	No
IO46	Unknown	α	Indian Ocean	-	Clokie *et al.*2006	No
IO47	Unknown	α	Indian Ocean	-	Clokie *et al.*2006	No
IO48	Unknown	α	Indian Ocean	-	Clokie *et al.*2006	No
IO49	Unknown	α	Indian Ocean	+	Clokie *et al.*2006	No
IO50	Unknown	α	Indian Ocean	+	Clokie *et al.*2006	Yes
S-IO8	Unknown	WH7803	Indian Ocean	-	Clokie *et al.*2006	No
S-IO8D	Unknown	WH7803	Indian Ocean	-	Clokie *et al.*2006	No
S-IO9B	Unknown	WH7803	Indian Ocean	-	Clokie *et al.*2006	No
S-IO28D	Unknown	WH7803	Indian Ocean	-	Clokie *et al.*2006	No
S-IO36	Unknown	WH7803	Indian Ocean	+	Clokie *et al.*2006	No
S-IO41	Unknown	WH7803	Indian Ocean	+	Clokie *et al.*2006	No
S-IO80	Unknown	WH7804	Indian Ocean	+	Clokie *et al.*2006	No
S-RSMI	*Myoviridae*	WH7803	Red sea	-	Millard and Mann. 2006	No
RS5	*Myoviridae*	WH7803	Red sea	-	Millard and Mann. 2006	No
RS9			Red sea	+	Millard and Mann. 2006	Yes
RS11	*Myoviridae*	WH7803	Red sea	+	Millard and Mann. 2006	Yes
RS14	*Myoviridae*	WH7803	Red sea	-	Millard and Mann. 2006	No
RS18			Red sea	+	Millard and Mann. 2006	Yes
RS20			Red sea	-	Millard and Mann. 2006	No
RS22			Red sea	-	Millard and Mann. 2006	No
RS23			Red sea	+	Millard and Mann. 2006	Yes
RS26	*Myoviridae*	WH7803	Red sea	+	Millard and Mann. 2006	Yes
RS27			Red sea	+	Millard and Mann. 2006	Yes
RS30			Red sea	+	Millard and Mann. 2006	No
RS32			Red sea	+	Millard and Mann. 2006	Yes
RS37			Red sea	+	Millard and Mann. 2006	Yes
RS38			Red sea	+	Millard and Mann. 2006	Yes
RS39	*Myoviridae*	WH7803	Red sea	+	Millard and Mann. 2006	No
RS44			Red sea	+	Millard and Mann. 2006	Yes
RS45			Red sea	+	Millard and Mann. 2006	No
RS49			Red sea	+	Millard and Mann. 2006	No
RS51			Red sea	+	Millard and Mann. 2006	No
RS53			Red sea	+	Millard and Mann. 2006	No
RS56			Red sea	+	Millard and Mann. 2006	Yes
RS57			Red sea	-	Millard and Mann. 2006	No
RS60			Red sea	-	Millard and Mann. 2006	No
RS62			Red sea	-	Millard and Mann. 2006	No
RS67			Red sea	-	Millard and Mann. 2006	No
RS68			Red sea	-	Millard and Mann. 2006	No
RS76			Red sea	-	Millard and Mann. 2006	No
RS81			Red sea	+	Millard and Mann. 2006	No
RS84			Red sea	-	Millard and Mann. 2006	Yes
RS85	*Myoviridae*	WH7803	Red sea	+	Millard and Mann. 2006	Yes
Bourget phage	*Myoviridae*	α	Lake Bourget, France	+	γ	Yes

α Yet to be screened for host range

γ Stéphan Jacquet, personal communication

Translation implies that the sequence could be translated into an open reading frame using the translate tool on ExPASy.

Phylogenetic analysis was then performed on the 16 phages from Group 1, Figure 2. We also included the Clade A and Clade B bacterial *mazG* sequences and phage *mazG* sequences available in NCBI, specifically Mx8, R.S101, M-L5, P-SMM4, P-SMM2 and Syn9. All 16 phages from Group 1 formed a tight group with no clustering by isolation site, suggesting that the sequences are of similar evolutionary origin despite the fact that these phages originate from very different geographical locations. This implies that the *mazG* gene is either a recent acquisition to the cyanophage population or that the effective population size is very small and the population cannot sustain high diversity. Furthermore, these sequences had only one translatable reading frame, stop codons being present in the other two frames. The tree also suggests that an ancestor of S-PM2 acquired its *mazG* gene from an organism similar to *Chloroflexus aurantiacus,* rather than a *Synechococcus* host (Figure 2).

Like S-PM2, the other 15 Group 1 phages can also infect *Synechococcus sp* WH7803 and other marine *Synechococcus* strains, while their ancestor probably acquired its *mazG* gene from an organism similar to *C. aurantiacus.* Furthermore, *mazG* phage sequences obtained from the database for P-SMM4, P-SSM2 and Syn9, (which infect *Prochlorococcus* strains and *Synechococcus* strains, respectively), also grouped with *C. aurantiacus* and not their respective hosts, Figure 2. This trend was also seen with the terrestrial and marine phages obtained from the database, R.S101, M-L5 and Mx8, which infect *Roseobacter, Mycobacterium* and *M. xanthus,* respectively. These phages did not directly group with the cyanophages (Figure 2), but were still closely related to these phages since they group with *mazG* Clade B. This suggests that their *mazG* gene is potentially more closely related to marine cyanobacteria and bacilli than to their hosts in Clade A (Figure 1). These basic topological features were found to be robust to alignment and under sequence truncation to the primer region, data not shown.

**Figure 4:**
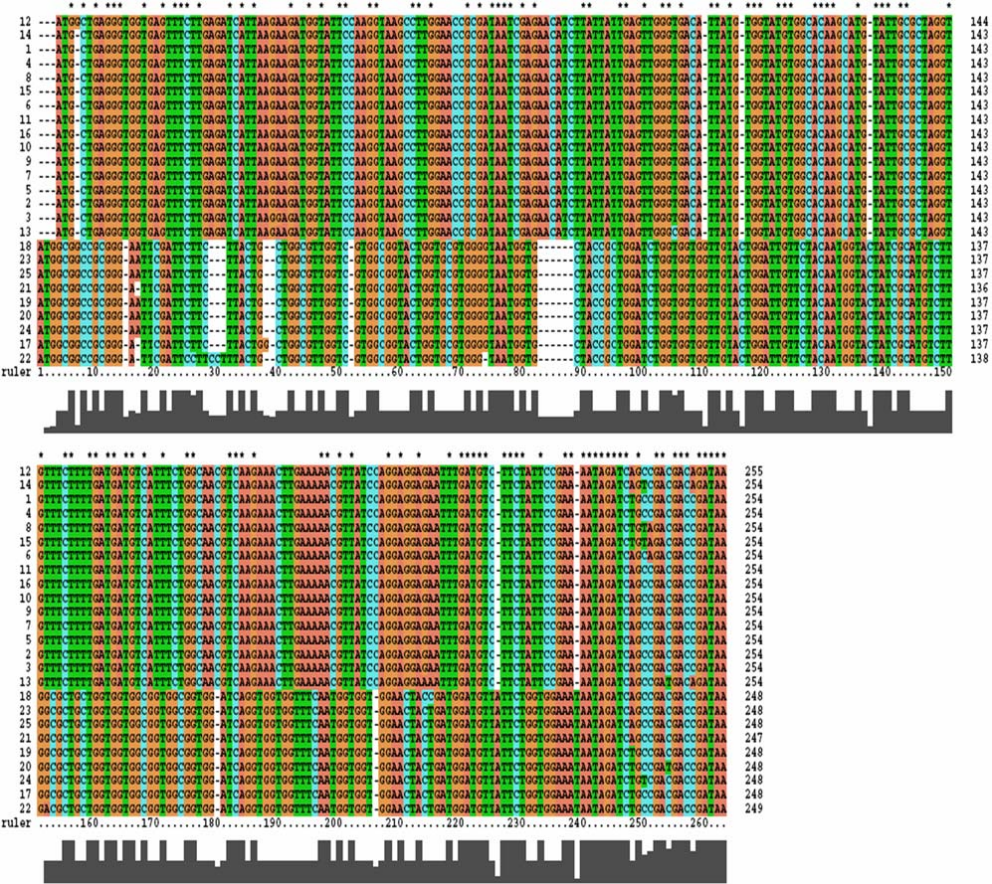
Nucleotide sequence alignment of the Group 1 and Group 2 phages. Group 1 phages are numbered 1–16, SMM1, S-BP3, RS18, RS11, S-IO41, SBnM1, S-PM2, RS27, SMM5, RS37, RS23, RS56, FWP, RS9, RS26 and IO50 and Group 2 phage sequences 17–25, RS85, RS39, IO39, RS38, IO15, IO41, IO17, S-IO80 and IO18. Both Group 1 and Group 2 phage sequences align closely within their own groups while showing some sequence similarity at the C-terminus.

The remaining 26 phages produced a PCR product of 279 bp and when this was sequenced and analysed using BLAST and BlastP, only 9 phages (IO41, RS85, IO39, RS39, IO15, S-IO80, IO18, RS38 and IO17), designated **Group 2**, showed some similarity to the S-PM2 *mazG* gene at the C terminal region. However, MotifScan was unable to detect the presence of a MazG domain. These phages were also included in the phylogenetic analysis (Figure 3), and grouped with the Group 1 phages, although the branch length separating them from Group 1 was over 1.0 (1.54). Examination of the alignments, Figure 4, shows that there is a partial homology with the *mazG* gene at the C terminus, with nucleotides showing some degree of conservation to the Group 1 phage. This explains their location as part of the Group 1 clade, while an unrelated N terminus results in an extended branch length. These sequences are however highly conserved within their own small group, despite coming from two different geographical locations (Indian Ocean and Red Sea), implying that this gene must provide a selective advantage, suggesting it may play an essential role in phage biology. Interestingly, Group 2, as with Group 1, probably acquired this sequence recently from a single ancestor, since there is a high level of conservation in these sequences; in fact all are practically identical.

**Figure 5:**
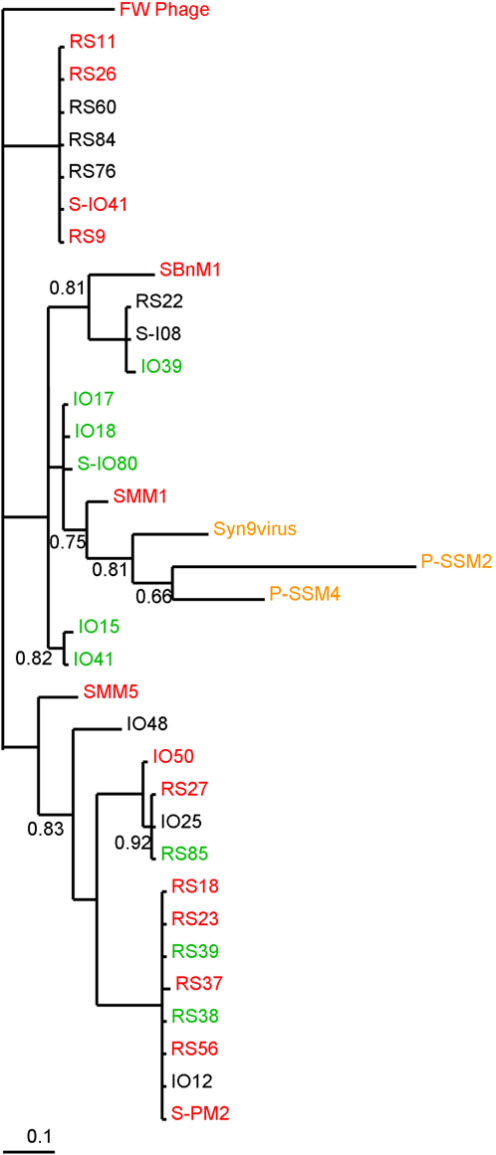
Consensus phylogenetic tree of the portal gene from both sequenced phages and phages isolated from different geographical locations. Group 1, Group 2 and database phage sequences as in Figures 2, 3. The non-*mazG* group, RS60, RS84, RS76, S-IO8, 1048, RS22, IO25 and IO12 comprise phage sequences for which our primers failed to detect *mazG.* Trees are unrooted and were generated from DNA codon alignments. Clade support values are shown at the nodes of the clades, only values below 0.95 are shown. Group 1 and Group 2 phage are coloured red and green respectively with phage sequences from NCBI in orange, as in Figure 3. The non-*mazG* phage group are coloured black.

To determine whether the *mazG* gene has undergone lateral gene transfer we compared the *mazG* phylogeny with that of a portal gene, using primers that generate a 165 bp product as described in Fuller *et al.,* 1998 [12]. We sequenced the portal gene for the Group 1and Group 2 phages, as well as eight additional phages (RS60, RS84, RS76, S-IO8, 1O48, RS22, IO25 and IO12), for which we were unable to detect a PCR product for the *mazG* gene, Table 2. In the following we refer to the latter as the non-*mazG* phage group. Phylogenetic analysis was then performed on these sequences together with portal gene sequences from the cyanophage Syn9, P-SSM2 and P-SMM4. The tight groupings previously observed for *mazG* are lost, specifically Groups 1, 2 and the non-*mazG* group intermix, with only the clustering of Syn9, P-SSM2 and P-SMM4 being retained. Group 1 and Group 2 phages are more divergent than in the *mazG* gene, and in particular, group approximately by geographical region with two Red Sea clades, an Indian Ocean clade and the fresh water phage being an isolated outgroup (Figure 5). This implies that the Group 1 (and Group 2) isolates are heterogeneous with respect to the portal gene.

To determine whether the *mazG* gene is under significant selection, we estimated the ratio of nonsynonymous/synonymous evolution rates (dN/dS). This reveals that the *mazG* gene in the Group 1 phages is under constrained protein evolution, average dN/dS = 0.27, similar to that of the other *mazG* phage genes (Mx8, L5, P-SSM2, P-SSM4, Syn9, R.SI01) with dN/dS = 0.24, and the Clade A bacterial *mazG*, average dN/dS = 0.35. Constraints are weaker than on the portal gene as expected, average dN/dS = 0.07 on the Group 1 portal gene; the portal gene being under high protein conservation constraints. The evolutionary time between Group 1 phages for the portal gene is approximately 3 times longer than that for the *mazG* gene, this is despite the fact that the *mazG* gene is in only 18% of cyanophages whilst the portal gene is ubiquitous in the myoviridae. Thus, there is no evidence of differential selective pressure on the Group 1 isolates, as might have been expected of a recently laterally transferred gene to a new environment.

The remaining 17 phage PCR products where analysed using Blast and ExPasy; 9 out of the 17 phages showed no similarity to any sequences in the NCBI database. The remaining eight sequences showed similarities to known database genes. These included a flavin containing monooxygenase, g20 from *Synechococcus* phage P6, gp19 a T4-like tail tube protein from the cyanophage P-SMM4, the *car* gene cluster from *Pseudomonas resinovorans* CA10, and *menF* from *E. coli,* the latter being an isochorismate synthase.

## Discussion

This study has demonstrated that the *mazG* gene is widely distributed with approximately 18% of cyanophages possessing the gene across diverse geographical locations, including the Red Sea, the Indian Ocean, the North Sea, Bermuda, the Atlantic Ocean, the Gulf of Mexico and Lake Bourget in France (freshwater). Despite this geographical diversity, we demonstrated that the phage *mazG* gene is highly conserved in the phage population at the DNA level. Thus, there is clear evidence to suggest that this gene is important in the lifecycle of these phages and it provides a distinct selective advantage.

CLUSTAL X alignment revealed conserved regions in the nucleotide sequence of the Group 1 phages, which matched bacteria and terrestrial phage sequences taken from the NCBI database. The alignment profile and the positive identification of a MazG domain by MotifScan, CDART and SMART, implies that these conserved regions encode key amino acid residues in the pyrophosphohydrolase, most likely the active site of the MazG protein. Similar results have previously been reported [13], [14], implying that highly conserved host-like genes are not uncommon in environmental phages and therefore that the total diversity of the global phage genomic pool could be significantly smaller than an assessment of phage gene content would indicate. The presence of a nearly identical gene sequence to ORF136 (*mazG*) from S-PM2 in S-BP3 (a member of the podoviridae) indicates that both podoviruses and myoviruses have access to the same gene pool and can freely exchange genetic elements by homologous recombination during co-infection.

Only a small number of phages were analysed in this study compared to the total diversity of the viriosphere, but our results suggest that ORF136 (*mazG*) of S-PM2 is widely distributed among both myoviruses and podoviruses in diverse geographical locations. Phylogenetic analysis demonstrates that marine phage sequences form a separate clade to their cyanobacterial hosts, clustering more closely with *C. aurantiacus,* and suggesting that the ancestral acquisition of this gene by a marine phage was more likely to have occurred during the infection of a *Chloroflexus* relative and not a *Synechococcus* strain. We have not been able to ascertain the ancestral host in this study, but based on the high conservation of this gene we expect that there will be a very similar bacterial gene in the environment. *C. aurantiacus* is a filamentous photosynthetic bacterium often found in hot springs. It is distinct from *Prochlorococcus* and *Synechococcus* as it fixes carbon by the 3-hydropropionate pathway and is therefore considered to be the most phylogenetically ancient of the anoxygenic phototrophs, which diverged early on in the evolution of the domain Bacteria [15]. Interestingly, members of the Chlorofexi-related SAR202 cluster are found universally in geothermal, soil, freshwater, marine and subsurface environments [16]. SAR202 cluster organisms occur throughout the mesopelagic zone underlying the photic zone; the photic zone being rich in cyanobacteria and their phage [16]. The coexistence between cyanophages and cyanobacteria suggests that some cyanophages, either historically or currently, were able to utilise both bacterial genera as a host and this may have led to the horizontal transfer of the *mazG* gene from a *C. aurantiacus* relative, most-likely a SAR202 cluster organism, to a *Synechococcus* phage. This may then have been widely distributed in the phage population through homologous recombination between phages within a shared network of hosts.

For the sequences represented by Group 2 (IO41, RS85, IO39, RS39, IO15, S-IO80, IO18, RS38 and IO17), only a small part of the C-terminal end of the sequence matched the *mazG* sequence from S-PM2. Although the phylogenetic analysis suggests that this gene is not *mazG*, it is well conserved across all 9 phages. We were not able to ascertain the origin of this partial *mazG* gene from this study, or the role of this gene in these phages. Once again, geographically distinct phages have retained a highly conserved gene. Out of the remaining 17 phage sequences detected via PCR, only 8 showed homology to any genes in the database. The majority of these genes could be translated, but it remains unclear whether they are indeed expressed in these phages, or what selective advantage they provide.

The fact that the MazG sequence from the Mycobacteria phage L5 is more closely related to *Synechococcus sp* WH8102 , *Prochlorococcus sp*. NAT12A and *Prochlorococcus sp*. SS120 sequences than its host, suggests that phages in all environments have access to a common gene pool. The Mx8 phage also does not group with its host *M. xanthus,* instead grouping with the more diverse *mazG* clade, Clade B, comprising marine and terrestrial bacteria. The Roseobacter phage R.S101 also groups with *Synechococcus sp* WH8102, *Prochlorococcus sp*. NAT12A and *Prochlorococcus sp*. SS120 instead of its host *Roseobacter*. This suggests that the *mazG* gene in these phages may not have been acquired from their primary hosts, and perhaps at some stage these particular phages may have infected marine cyanobacteria or alternatively acquired their *mazG* gene from a common host.

The high prevalence and conservation of the *mazG* gene amongst our isolates at the DNA level across diverse geographical locations indicates that this gene provides a significant selective advantage to the phage. However to ascertain if the *mazG* gene alone or the phage are the unit of selection it is necessary to determine whether the Group 1 phages are distinct as a group for other genes. This is in fact not the case; a phylogenetic analysis of the portal protein shows that the groupings of the phage isolates in the *mazG* phylogeny by presence of a MazG domain (Group 1) and its absence (Group 2) is completely lost in the portal gene, and in particular, group approximately by geographical region with two Red Sea clades, an Indian Ocean clade and the fresh water phage being an isolated outgroup (Figure 5). Other phage isolates, which do not contain the *mazG* gene, also intermix implying that the Group 1 (or Group 2) isolates are a heterogeneous group for the portal genes. This strongly supports the hypothesis of promiscuous gene transfer between phages suggested by Hendrix *et al*., [17].

Hendrix *et al*. proposed a model in which all of the dsDNA phages and prophage genomes are mosaics with access via lateral transfer to a large common gene pool. They suggested that this access was not uniform, with phylogenetically local areas of free and intense exchange coupled with exchange beyond the constraints of the local neighbourhood at a reduced frequency. Thus, any phages can have access to all of the sequences in the global pool, but the frequency of this access depends on the number of barriers (*e.g*. host range) between the phage and any particular sequence and, therefore, how many individual steps of genetic exchange are required to bring them together. The high prevalence of the *mazG* gene reported here (18% with MazG domain) indicates that the viral gene pool is globally connected on an (evolutionary) rapid time scale, as indicated by the lack of geographical isolation of the *mazG* ^+^ phages. It is possible that *mazG* acquisition is ongoing and a higher fraction of cyanophages will ultimately acquire the *mazG* gene. This hinges on whether the 18% of cyanophages that currently have the *mazG* gene are a distinct subpopulation of cyanophages that are, both, highly connected to the phage gene pool and have a selective advantage upon acquisition of the *mazG* gene. The fact that we failed to detect differential selective pressure on the *mazG* gene between the Group 1 isolates, the Clade A bacterial *mazG* gene and database phage sequences (Mx8, L5, P-SSM2, P-SSM4, Syn9, R.SI01), suggests that it is not under significant adaptive evolutionary pressure, despite the fact that its diverse spread indicates that it provides a significant advantage to the phage. Thus, it is more likely that these cyanophage are a distinct subpopulation. We hypothesise that the *mazG* gene is under intense exchange within the cyanophage population, resulting in a small effective population size, thereby explaining the high levels of sequence similarity as a consequence of a recent common ancestor. This compares to the portal gene, which has a higher effective population size, and thus higher sequence diversity. Our study thus indicates that there is a high level of disparity between levels of exchange of individual genes.

The presence of host like genes, such as the *mazG* gene, in phages is a frequent occurrence but by no means a default component of all cyanophage genomes. The reason for the presence of the *mazG* gene in some but not all phages could be due to phage behaviour during infection. It has been suggested that phages with a shorter latent period are less likely to possess host-like genes that perturb metabolism, as they will not benefit from their expression due to the short time spent in the host cell. For example, *psbA,* has not been found in phages that have a latent period shorter than 8 hours [18]. Phages with a longer latent period will need to keep the cell alive to ensure optimal burst size, and may acquire host genes in order to do this. In S-PM2, *mazG* lies under the control of a putative early promoter [2]. This could imply that this gene is responsible for adapting host metabolism to create a suitable environment for maximal phage replication, similar to the function of *mazG* in *E. coli* [5]. The virally encoded *mazG* could have a role in restoring protein synthesis in a starved cell to allow for virus replication. This is a feature that phages in nutritionally deplete environments would find beneficial, thus explaining the apparent ubiquity of this gene in the marine and terrestrial phage population. Whether the other phages possessing *mazG* found in this study also express this gene early during their infection cycle remains to be elucidated.

## Materials and Methods

### DNA Extraction

Phage isolates were obtained from a variety of sources as indicated in Table 1. 1 ml of lysate for each phage was treated overnight with 5 µl DNAase (Promega), according to the manufacturer's specifications. This was then extracted in 1 volume of phenol, followed by vortexing and centrifugation for 2 minutes at 15 000 g in a bench top microcentrifuge. The aqueous layer was then extracted twice with 1 volume 24∶23∶1 phenol/chloroform/iso-amyl alcohol and centrifuged as above. DNA was precipitated by the addition of 1/10 volume 3 M sodium acetate and 1 volume propan-2-ol for one hour on ice. Following centrifugation at 15 000 g for 20 minutes the pellet was re-dissolved in 50 µl of TE (Tris/EDTA) buffer.

### Primer design

Both nucleotide and protein sequences for ORF136 of S-PM2 were used to identify similar sequences in the NCBI database by BLAST searching (http://www.ncbi.nlm.nih.gov/BLAST/), using an E-value cut off of 10^−18^. Phage nucleotide sequences were aligned using CLUSTAL X (version 1.81). Primers were designed using Primer Designer^©^ 3.0 (Scientific and Educational Software, Durham, N.C.). Primers were synthesised commercially by VH Bio (Gateshead); forward primer: 5′ CTT CTT ACT GCT GSY GTT GG 3′, reverse primer: 5′ TTA TCK GTC RTC KRC WGA TT 3′. Primers were designed internally to the ORF136 gene (total length 438 bp) generating a PCR product of 279 bp (64% of total gene sequence). The portal gene was amplified using the primers CPS1 (5′-GTAG[T/A]ATTTTCTACATTGA[C/T]GTTGG-3′) and CPS2 (5′-GGTA[G/A]CCAGAAATC[C/T]TC[C/A]AGCAT-3′) as described in Fuller *et al*., 1998.

### Touch-up PCR, Gel Extraction and pGEM Cloning

5 µl of template DNA (Table 1) was added to 25 µl Promega master mix (Promega®) with 0.5 µl of each primer (1 nm) and 19 µl distilled water. PCR conditions: initial denaturation at 95°C for 1 min, followed by 15 cycles of denaturation at 95°C for 30 s, annealing at 45°C for 30 s and extension at 72°C for 30 s, and 15 cycles of 95°C for 15 s, 55°C for 30 s and 72°C for 30 s followed by a final extension at 72°C for 5 mins in a PCR thermal cycler (eppendorf). Samples were then analysed on a 1% agarose gel. Products were excised and purified using a QIAquick® gel extraction kit (Qiagen) according to the manufacturer's instructions.

Gel extracted samples were ligated into a pGEM®-T easy plasmid (Promega) according to manufacturer's guidelines. Plasmids were then transformed into competent *E. coli DH5α* cells (*LacZ^−^)* and spread onto Luria Broth Agar plates containing 5-bromo-4-chloro-3-indolyl-β-D-galactoside (X-Gal) and ampicillin. These were incubated overnight at 37°C. White colonies where picked onto fresh agar plates containing ampicillin and grown overnight at 37°C. Colonies were then used to inoculate LB broth containing ampicillin and incubated overnight at 37°C. Plasmids were then purified using the QIAprep® Spin Miniprep Kit (Qiagen) according to the manufacturer's instructions.

### Sequencing

For the sequencing reactions, 1 µg of plasmid DNA was added to 5 pmole of the Promega T7 primer and the total volume was made up to 6 µl with nuclease-free water. The sequencing was carried out at the Warwick University Molecular Biology Service.

### Computational Analysis of Sequence Data

Sequences were individually compared against GenBank using BLASTN and protein-protein Blast (http://www.ncbi.nlm.nih.gov/) to confirm their identity. Each sequence was also translated using the translate tool in ExPASy (http://www.expasy.org/). MazG motifs were identified using CDART (NCBI), MotifScan and SMART (ExPASy). Host bacterial nucleotide sequences and other non-marine viral sequences with significant E-values were extracted from KEGG (http://www.genome.jp/kegg/), CyanoBase (http://bacteria.kazusa.or.jp/cyano/) and NCBI.

### Phylogenetic analysis

*mazG* nucleotide sequences were aligned using ClustalW (http://www.ebi.ac.uk/clustalw), specifically, amino acid sequences were aligned with ClustalW and then the corresponding DNA alignment was determined by reverse translation using the original DNA sequences. Phylogeny was inferred using the Markov chain Monte Carlo package Mr Bayes (http://mrbayes.csit.fsu.edu/) with the HKY model that is parametrised by nucleotide frequencies, branch lengths and the transition-transversion ratio Κ. Convergence was confirmed using a multiple run methodology [19].
